# Does Skin Color Influence Differential Diagnosis Generation in a Simulated Cardiac Arrest?

**DOI:** 10.5811/westjem.53204

**Published:** 2026-06-28

**Authors:** Hannah E. Miller, Claudia Tarrant, Miles Lamberson, Cate Nicholas, Katherine Dolbec

**Affiliations:** * University of Vermont Health, Robert Larner College of Medicine, Department of Emergency Medicine, Burlington, Vermont; † University of Vermont, Robert Larner College of Medicine, Burlington, Vermont

## Abstract

**Introduction:**

It is not known whether skin color influences inclusion of substance use on medical students’ differential diagnoses for a simulated cardiac arrest. Our objective in this study was to investigate whether the presence of a dark-skinned or light-skinned manikin in the lab correlated with their decision to include substance use on the differential as a possible cause of cardiac arrest.

**Methods:**

In this study at a single institution, all fourth-year medical students in an emergency medicine course participated in a manikin-based cardiac arrest simulation case. The simulation sessions alternated between the use of a light-skinned and a dark-skinned manikin. Both were adult sized. After the first shock was delivered, students individually documented their differential diagnosis for the cause of the cardiac arrest on deidentified, free-text research forms. We excluded forms if they were blank, had been completed by a non-medical student, or could not be associated with the skin color of the manikin used. The primary outcome measure was how often students included substance use as a cause of cardiac arrest anywhere in their differential diagnoses. We compared the relationship between manikin skin color and the students’ consideration of substance use as a cause of cardiac arrest, using the Fisher exact test.

**Results:**

Of 270 eligible participants, 271 surveys were returned; 75 (27.7%) were excluded. Of the remaining 196 surveys, 96 were associated with the light-skinned and 100 with the dark-skinned manikin. Among the 100 respondents using the dark-skinned manikin, one student (1%) listed substance use as the leading diagnosis, while none (0%) of the students using the light-skinned manikin listed substance use as the leading diagnosis. In each group, only 20% of students (*n* = 39 total) listed substance use anywhere on the differential diagnosis (*P* = 1.00).

**Conclusion:**

Among fourth-year medical students participating in a simulated cardiac arrest, we found no correlation between manikin skin color and the inclusion of substance use on their differential for cardiac arrest. Overall, their decision to include substance use in their differentials was low regardless of skin color, suggesting the need to improve their awareness of substance use as a potential cause of cardiac arrest.

## INTRODUCTION

Drug overdose deaths are a leading cause of mortality in the United States. There were nearly 108,000 drug overdose-related deaths in 2022, according to the National Institutes of Health.^1^ In 2023 the U.S. Centers for Disease Control and Prevention’s State Unintentional Drug Overdose Reporting System reported victim demographics as follows: most were male (71.6%); 23% were Black, 61.3% were White; and 25.9% were 35–44 years of age.^2^ Of note, in recent years overdose death rates have increased disproportionately among non-Hispanic Black and non-Hispanic American Indian/Alaska Natives.^3^ Demographics describe the social milieu that helps inform how society constructs stereotypes of groups more likely to experience substance use.

Evidence is limited regarding the ways in which bias based on skin color affects clinical decision-making. Previous investigations of explicit and implicit bias in clinical practice were based on clinical case vignettes, rather than on actual human behavior in clinical scenarios (in situ or simulation-based).^4^ In those vignettes it was noted that participants tended to spend similar amounts of time on cases and create similar differential diagnoses regardless of patient presentation and/or background.^5^

We began this study at a time when racial inequities were discussed more frequently and race-based medical guidelines were being questioned and in flux. We believe that medical students have become more aware of and are more likely to consider the intersection between race and health outcomes and that their perceptions would influence their clinical practice. (Philips and Soltis describe this as the conceptual framework of knowledge construction and schemas.^6^)

Our objective was to investigate how often medical students included substance use on their differential diagnosis as a cause of cardiac arrest, depending on whether they interacted with a dark- or light-skinned manikin in the simulation lab. We hypothesized that students would be more likely to include substance use on their differential diagnosis regarding etiology of the cardiac arrest when performing the simulation on the darker-skinned manikin.

## METHODS

This was a prospective, double-arm experimental study performed at a single academic institution. Eligible for inclusion in the study were all fourth-year medical students enrolled in the four-week emergency medicine (EM) course, along with EM-bound visiting students from other medical schools. Demographically, most residents (91%) of our institution’s catchment area identify as White, while 24% of our medical students identify as being from groups that are under-represented in medicine.

Students received an information sheet describing the study’s purpose. They were given the choice to opt out of the study but still complete the required educational simulation case. They were blinded to the fact that the manikin’s skin color was relevant to the study. All enrolled students participated in a manikin-based simulation to learn the basic concepts of Advanced Cardiac Life Support (ACLS). Two adult-sized manikins (of no specific age or sex) with different skin colors but identical functionalities were used.^7^ The light- and dark-skinned manikins were alternated in each course section; thus, students were assigned to a manikin based on their course section enrollment. The case simulated a patient presenting as unresponsive and pulseless, with no contextual information provided. After the first shock was delivered, each student completed a deidentified research form with multiple blank spaces that allowed space to create an open-ended differential in order of likelihood, thereby limiting any leading bias.

**Population Health Research Capsule**

What do we already know about this issue?

*There is a knowledge gap of how bias based on skin color affects clinical decision-making*.

What was the research question?

*In a simulated cardiac arrest, would the manikin’s skin color influence medical students’ differential diagnosis?*

What was the major finding of the study?

*Whether the manikin was light- or dark-skinned, only 20% of students listed substance use as a cause of cardiac arrest on their differentials (P = 1.00)*.

How does this improve population health?

*Exploring the effect of racial bias, represented by skin color, on clinical decision-making could lead to more equitable healthcare*.

Research coordinators entered the data into a Research Electronic Data Capture (REDCap) electronic data capture tool hosted at the University of Vermont.^8^,^9^ The primary outcome was the proportion of cases in which, substance use was included on the differential diagnosis. We used the term “substance use” to encompass substance use, drug overdose, or anything related to a toxicologic etiology. The secondary outcome was whether the student’s primary diagnosis related to substance use. We grouped the data by manikin skin color. Data forms were excluded if they were left blank, had been filled out by a non-medical student, or if they could not be associated with a particular manikin.

We compared the outcome using a two-tailed Fisher exact test. A *P* value of .05 was used to determine significance. A study team member blinded to the manikin skin-color assignment completed data analyses We then conducted a post-hoc power analysis.

The study was deemed exempt by the Institutional Review Board.

## RESULTS

**Figure 1:**
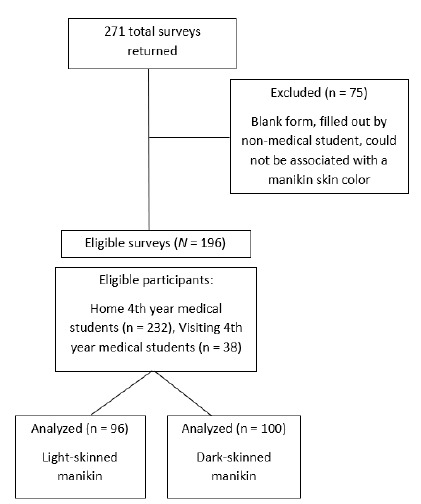
CONSORT-style flow diagram describing the population of medical students who participated in a simulated cardiac arrest case using dark- and light-skinned manikins. *CONSORT*, Consolidated Standards of Reporting Trials.

Eligible participants included 270 medical students, 38 of whom were visiting from other medical schools. A total of 271 surveys were returned (Figure 1), and 75 were excluded. Of the remaining 196 surveys completed during simulations, 96 were completed by students using the light-skinned manikin and 100 by those who used the dark-skinned manikin.

As shown in Table 1, 20% of the students in each group (N = 39 total) listed substance use somewhere on the differential diagnosis as a cause for the simulated patient’s cardiac arrest. A post-hoc power analysis demonstrated that the proportion of cases in which substance use was included on the differential diagnosis in the dark-skinned manikin group would need to be 38% for 80% power.

## DISCUSSION

In this study of fourth-year medical students participating in a simulated cardiac arrest case, we found no difference in their consideration of substance use as the etiology of the cardiac arrest based on manikin skin color. This study contributes to the medical education literature as there have been limited prior attempts to investigate whether there is a measurable effect of bias (predicated on the concept of knowledge constructivism) on clinical decision-making among healthcare professionals.^6^,^10^ Similar to the results of earlier published research, we found that manikin skin color did not affect the differential diagnosis.^5^ We chose skin color as the independent variable to indirectly communicate and test the concept of racial bias in the simulation environment. We acknowledge that skin color cannot be conflated with race; however, it does function as a cue to indicate social differences, given that various identities are associated with stereotypical external presentations.

Racism, rather than race itself, serves as the foundation for disparities. Health inequities are primarily driven by structural racial bias, the physical environment, health behaviors, and social determinants.^11^ Medical schools play an active role in propagating racial health bias. Researchers often rationalize racial group differences and perpetuate them as “scientific fact” during the production and propagation of knowledge. As a result, students construct mental maps that can bias clinical practice.^6^ Educational spaces are often regarded as racially neutral because whiteness is “invisible” (“color blind”) and presumed normative. Clinical training modules and lecture materials extensively use white bodies for clinical representations, thus serving as the norm with which topics are learned. Racism and racist ideologies are reified via the medical curriculum when students learn to associate race with phenotypes, behaviors, and diseases. Even cultural competency instruction tends to describe the practices of “other” cultural/racial groups as opposed to White culture, reinforcing the standard of White customs.^11^ This ultimately positions “others” as a deviation from the norm, creating subliminal content for construction of cognitive schemas that can lead to implicit and/or explicit bias.^6^

This study demonstrates a weak relationship between skin color and bias—or premature closure—when considering the potential causes of cardiac arrest in a patient. Of note, it is concerning how infrequently substance use was considered in the medical students’ differential diagnoses. It is important that they be taught to consider substance use when managing unresponsive patients and those in cardiac arrest, as drug overdose deaths are a significant source of mortality in the U.S. The American Heart Association (AHA) guidelines prioritize performing cardiopulmonary resuscitation (CPR) in cardiac arrest.^12^ As outlined in AHA algorithms, if components of CPR are not delayed, naloxone can be administered along with standard interventions if depressed respiration and/or opioid ingestion is suspected to have played a role.^13^,^14^ Future research could focus on medical students’ subjective experience within the simulation environment to elucidate the factors leading to their formulation of a differential diagnosis.

## LIMITATIONS

This study was completed at a single institution, limiting generalizability and external validation. The data collection form had an open-ended design, which led to inconsistencies in the data obtained. We did not control for medical student education on the topics of bias, race, or social disparities. This may have affected how students, particularly visiting students, interacted with the simulation experience. We excluded approximately six months’ worth of data because of the inability to match data forms with manikin color, leading to decreased power. The age of the manikin/simulated patient was not disclosed or evident, which may also have affected the differential diagnoses created by the students.

## CONCLUSION

Among fourth-year medical students who participated in a cardiac arrest simulation, we found no difference in their propensity to include substance use in their differential diagnoses, whether the manikin had dark- or light-colored skin. The overall rates of including substance use on their differentials for cardiac arrest were low (20%), regardless of manikin skin color, suggesting the need to increase student awareness of substance use as a possible cause of cardiac arrest.

## Figures and Tables

**Table 1:** Medical students’ differential diagnoses regarding the etiology of a simulated cardiac arrest in a study investigating whether manikin skin color influenced their perceptions.

	Total *N* =196	Light-skinned Manikin *n* = 96	Dark-skinned Manikin *n* =100	*P* value	Relative Risk (95% confidence interval)
Primary Diagnosis related to SU	1% (1)	0% (0)	1% (1)	1.00	Undefined
Any differential diagnosis includes SU	20% (39)	20% (19)	20% (20)	1.00	1.01 (0.58, 1.77)
SU Absent from Differential	80% (157)	80% (77)	80% (80)	1.00	1.00 (0.87, 1.15)

*SU*, substance use.
